# Factors Influencing Health Knowledge and Behaviors among the Elderly in Rural China

**DOI:** 10.3390/ijerph13100975

**Published:** 2016-09-30

**Authors:** Zhifei He, Zhaohui Cheng, Tian Shao, Chunyan Liu, Piaopiao Shao, Ghose Bishwajit, Da Feng, Zhanchun Feng

**Affiliations:** School of Medicine and Health Management, Tongji Medical College, Huazhong University of Science & Technology, Wuhan 430030, China; houis123@163.com (Z.H.); chengzhaohui@hust.edu.cn (Z.C.); shaotian111@foxmail.com (T.S.); chunyan_liu93@163.com (C.L.); spphust1993@sina.com (P.S.); brammaputram@gmail.com (G.B.); fdnunu@163.com (D.F.)

**Keywords:** health knowledge, health behavior, influence factors, elderly people

## Abstract

Objectives: Health knowledge and behaviors are the key elements that ensure high quality of health for the elderly. This study explored and determined the conditions and factors of health knowledge and behaviors that affect the elderly in rural China. Methods: A cross-sectional research approach and random stratified sampling method were used in 12 towns and 48 villages in the Chongqing Municipality, Henan, and Zhejiang Provinces in China from June to September 2013. The collected data included: (1) socio-demographic characteristics of 1593 elderly people; (2) accuracy rate on health knowledge of the elderly, which was analyzed and compared among the three sample areas by using Chi-square test; and (3) mean scores on the health behaviors of the elderly, which were analyzed and compared by using analysis of variance (ANOVA). The multiple-linear regression method was used to analyze the factors affecting the health knowledge and behaviors of the elderly. Results: Significant differences were observed among the nine items in the health knowledge questionnaire (*p* = 0.000 < 0.001). The average accuracy rate of the nine items was 57.43%. Significant differences were observed among the eleven items on the health behaviors of the elderly in the sample rural areas (*p* = 0.000 < 0.001). Age, economic level, degree of education, distance from home to medical institutions and disposable personal income (DPI) can affect the scores of the health knowledge and behaviors of the elderly (*p* = 0.000 < 0.001). Conclusions: Lack of health knowledge and poor health behaviors are common among the elderly in the sample areas of rural China. This deficiency poses a serious threat on the promotion of health conditions and the improvement of the level of health quality among the elderly. Different types of access to sources of health knowledge should be used to increase health knowledge scores of the elderly. Various potential intervening measures should also be adopted to improve their health behaviors of elderly people.

## 1. Introduction

Health literacy is defined as “the degree to which individuals have the capacity to obtain, process, and understand basic information and services needed to make appropriate decisions regarding their health” [1]. Health literacy is a relatively new concept that has gained significant interest over the past decade. This concept has been accompanied by an increased emphasis on the role and responsibility of citizens regarding health-related issues [2,3,4]. Health knowledge and behaviors are two important elements of health literacy. These elements have a significant effect on the daily lives of residents, particularly the elderly. 

Previous studies on health knowledge showed how and whether a person obtains health information can affect their health behavior, healthcare access, health outcomes, and quality of life. A related study on the factors affecting the advancement of health knowledge and behaviors of the elderly may be caused by their awareness about their health, to a certain extent [5]. A previous study determined that health knowledge remains an important goal of health education programs for residents of China [6]. Another study determined a low socio-economic status is closely related to an individual’s educational level [7,8]. Adults who lack formal education have a high probability of unemployment, which results less income, thereby leading to poor quality of health [9]. Health promotion is based on health education, and health knowledge is the foundation of health education [10]. The rate of the health literacy level of Chinese residents was 6.48%, which indicates that seven out of 100 people have poor health literacy [11].

According to an international study [12], a person’s health behavior includes intentional activities that aim to protect or improve one’s health. However, a healthy person has a different health goal as compared with a sick individual. Thus, an individual’s health should be considered in terms of the symptoms that one’s experiences, the intensification of symptoms, and how the symptoms affect an individual’s function in different areas of his life [13]. The effectiveness of health education depends on people’s beliefs regarding the importance of new information and on their confidence in their ability to change their own health behaviors [14]. The improvement of the health knowledge and behaviors of the elderly is also helpful in strengthening their ability to carry out practical treatment for their illness, promoting the rational use of existing medical and health resources, enhancing their awareness of disease prevention and self-health care, and enabling them to make correct judgments for their own health as well as when dealing with public health emergencies scientifically [15]. 

Health knowledge is linked to the awareness, motivation, and competence of people in accessing, understanding, appraising, and applying health information. These factors help to maintain or improve individuals’ behaviors and quality of life by making the appropriate judgments and forming decisions regarding healthcare disease prevention and health promotion in their daily lives. A simultaneous relationship between the reasonable evaluation of health demand and improving the health behavior and health levels of the elderly was observed. Furthermore, enrollees with chronic illnesses, who possessed increased rates of low health knowledge, lacked proper health behaviors. Increasing evidence has shown that the lack of health knowledge and health behaviors are associated with problems connected with the use of preventive services, delayed diagnoses, understanding one’s medical condition, one’s adherence to medical instructions, self-management skills [16], and health outcomes. Another study [17,18] determined that the elderly who lack knowledgeable about diseases and ignore the proper health behaviors, cannot sufficiently understand the need for preventive care. 

In many ways, aging in developing countries is more difficult than that in developed countries. According to China’s sixth census, more than 119 million people in the country are 65 years old or older, accounting for 8.89% of the population. Demographers project that by 2050, the number of people aged 65 and above will reach approximately 400 million. Currently, approximately 70% of people older than 65 years of age live in rural areas [19]. In particular, the rate of rural population aging is higher than that of urban population aging, which has exceeded 15.4%. The social economic status and degree of health literacy of the rural population are lower than the urban population [20]. Some foreign studies have also shown different degrees of health literacy in different areas. Griffiths et al., selected the outer regional, remote, and very remote areas of Australia to study the health literacy of its residents [21]. Kondilis et al. examined and compared the research productivity on selected fields related to the health literacy of current members of the European Union [22].

The literature reveals that in the 1980s, approximately 80% of the American population aged 65 and above suffered from one chronic disease because they lacked the necessary health knowledge and basic health behavior. Because of the low educational degree and poor education quality in rural areas, the majority of the elderly lacked health knowledge, health awareness, and the accompanying health behaviors. A previous study showed that almost two-thirds of the elderly people in rural Sichuan Province, China were not aware of what normal blood pressure was and how hypertension can be prevented [23,24]. 

Researchers have conducted many studies on the health literacy of the elderly with high blood pressure in Chinese urban areas [25,26], and on the mental health literacy of young people abroad [27,28,29]. In China, most studies focused on the health knowledge and health behaviors of the elderly in urban areas, as well as on the alternative health knowledge or relationships between human behavior and health outcomes [30]. Other studies have focused on a certain behavior, such as smoking, drinking, and physical exercises that affect people’s health, but lack information regarding the multiple factors that influence one’s health status. However, few scholars have focused on the health knowledge and behaviors as well as the associated factors that affect the health quality of the elderly in rural areas. Thus, the present study aims to explore the related factors that affect and will further improve the health knowledge and behaviors of the elderly in the sample areas in rural China, and to fill the observed gap [31]. 

## 2. Methods

### 2.1. Study Design

A cross-sectional research approach and a random stratified sampling method were used to analyze the health knowledge (the total items about the elderly’s common sense in health), health behaviors, and factors that affect the health knowledge and behaviors in sample areas. This study was conducted from June to September 2013. 

The questionnaire used in this study was self-designed and based on widely available literature. This questionnaire was composed of three parts, namely, socio-demographic characteristics of the elderly in sample areas, their health knowledge, and their health behavior. Trained and qualified investigators were sent to each sample area during the survey period. A team of two investigators were tasked to create a face-to-face survey for the elderly in the sample villages. The village doctor first collected information from the elderly according to our survey requirements, and then our investigators personally spoke with the survey participants. The completed questionnaires were collected and checked at the survey location.

### 2.2. Structure Model

**Figure 1:**
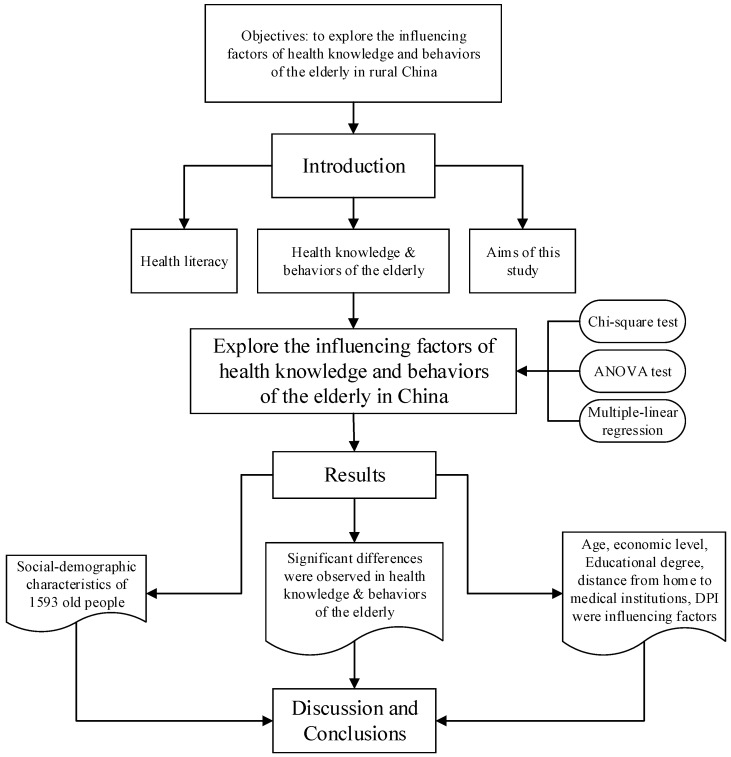
Framework of this research.

We provide a structure model of this research manuscript (Figure 1) to explain and state the idea of our writing. By the literature study and field investigation, we explored the influence factors of health knowledge and behaviors of the elderly in rural China through Chi-square test, ANOVA test and multiple-linear regression. Then, we analyzed the results, discussions and got the conclusions.

### 2.3. Data Collection

A cross-sectional survey was conducted from June to September 2013 in Chongqing Municipality, Henan, and Zhejiang provinces in China, which corresponded to the Western, Central and Eastern areas respectively, in accordance with our previous project design (Table 1). The level of economic development was observed to increase from Chongqing, Henan and Zhejiang. A random stratified sampling survey was adopted. Four towns were chosen randomly from each sample municipality/province, four villages were selected from each town randomly, and 36 elderly people (with an age range of 60 years old and above) were selected from each village. Thus, 16 villages and 576 elderly people were selected from each sample municipality/province based on our study design. A total of 533, 522, and 538 detailed questionnaires with 92.53% effectiveness rate in Chongqing, 90.63% effectiveness rate in Henan, and 93.40% effectiveness rate in Zhejiang respectively, were obtained. In total, a number of 48 villages were selected, 1728 elderly people participated in the face to face survey, and 1593 responses were found to be valid in this study.

### 2.4. Variables

The questionnaire was composed of three parts. The part on socio-demographic characteristics included information on the individual’s age, gender, degree of education, profession, type of healthcare insurance, distance from home to medical institution, and disposable personal income (DPI). The health knowledge part included nine items, and two choices were provided for each item (i.e., yes and no). The part on health behavior included eleven items, with each item containing four different dimensions according to the Likert scale. Four kinds of scores represented the four different degrees for each item, namely, 0, 1, 2, and 3. A higher score obtained, indicated better health behavior of the elderly.

We personally designed the questionnaire based on some domestic and foreign literature [32,33,34,35,36]. All the variables were checked by professors after we completed designing questionnaire. Cronbach’s alpha coefficient of the entire questionnaire was 0.832, and the Cronbach’s alpha coefficient, which was higher than 0.7, was valid according to previous study results.

### 2.5. Statistical Analysis

Data entry was performed via Excel 2013 version (Microsoft Office, Redmond, WA, USA) and EpiData 3.1 (EpiData Association, Odense, Denmark). The double-entry method was adopted to ensure data accuracy, and SPSS 19.0 statistical software (IBM Company, Armonk, NY, USA) was used for the statistical analysis. The socio-demographic characteristics of the elderly were described by using the descriptive statistics method. Chi-square test was adopted to analyze and compare the accuracy rates of the health knowledge of the elderly. ANOVA was used to compare the mean scores of the health behavior of the elderly in the sample areas, as well as the analysis of the mean scores of the health knowledge and health behaviors of the elderly under different socio-demographic conditions in the sample areas. Finally, a multiple-linear regression method was adopted to analyze the factors influencing the health knowledge and behaviors of the elderly.

### 2.6. Ethics Approval

The study protocol was approved by the Research Ethics Committee of Tongji Medical College, Huazhong University of Science and Technology (IRB No.: FWA00007307). All participants indicated their willingness to participate in this research.

## 3. Results

The results of the socio-demographic characteristics of the elderly in sample areas are shown in Table 2. This table shows that 1593 samples were valid. A total of 533, 522, and 538 of these samples came from Chongqing, Henan and Zhejiang, respectively.

A significant difference was observed among the age groups (*p* = 0.000 < 0.001). The largest age groups were that of 66–70 and 71–75 years old. A significant difference was observed between the genders (*p* = 0.000 < 0.001). Five types of educational degrees had significant differences (*p* = 0.002 < 0.01). In general, the number of samples based on educational attainment were observed to decrease gradually from being illiterate to having a college degree. Approximately half of the elderly people were illiterate, and less than 3% had college degrees. Significant differences were observed in the professions of the elderly (*p* = 0.000 < 0.001). The majority of the sample (74.63%) was farmers. The proportion of farmers was 92.68%, 62.84%, and 68.22% in Chongqing, Henan and Zhejiang, respectively. A small percentage of the elderly was engaged in four other professions. The elderly in the samples had three types of health care insurance, and a significant difference was observed among these types (*p* = 0.001 < 0.01). Three types of healthcare insurance were selected by elderly people, including New Cooperation Medical System (NCMS), Urban Residents’ Basic Medical Insurance (URBMI) and Urban Employee Basic Medical Insurance (UEBMI). More than 90% of rural elderly people chose the NCMS. A significant difference was observed between the two groups in terms of distance from home to medical institutions (*p* = 0.000 < 0.001). In general, more than 95% of elderly people lived near medical institutions, and the distance from their homes to medical institutions was less than 1 km. The results showed significant differences in the five types of DPI (*p* = 0.000 < 0.001). The DPI of less than 5000 RMB and more than 20,000 RMB were the largest rates among the five DPI groups (*p* = 0.000 < 0.001). In Zhejiang and Henan, more than 40% of the elderly had more than 20,000 RMB in DPI, whereas in Chongqing, only 12.75% of the elderly had the DPI of more than RMB 20,000 RMB.

The accuracy rate of health knowledge and mean scores of health behavior of the elderly in the sample areas are shown in Table 3. The results showed significant differences among the 9 items. Among these items, item 6, which asks “whether fruits can be eaten without washing” had generally higher accuracy rate than others (83.55%), whereas item 9, which asks “whether health is defined as not being fat or thin”, had the lowest accuracy rate (32.83%). The average accuracy rate of the 9 items was 57.43%. In general, the majority of the accuracy rates of health knowledge items increased, with Chongqing as the lowest, followed by Henan, and Zhejiang. However, among the nine items in the health knowledge, the accuracy rates of item 5, which asks “whether excessive drinking is harmful to the liver”, and item 6, which asks “whether the fruits can be eaten without washing”, were found to be lower in Henan than Chongqing.

In view of the scores of the health behaviors of the elderly, significant differences were observed among the questions, except for item 2, which asks for the “frequency of tooth brushing”. The results demonstrated that the mean score of health behavior in Zhejiang was higher than that in Henan and Chongqing. In item 5, which asks about “physical examination” and item 8, which asks about the “frequency of blood pressure measurement”, both the mean score of the health behavior of the elderly in Chongqing were higher than those in Henan. Nevertheless, in item 6, which asks about the “frequency of physical exercises” and item 11, which asks about the “frequency of disseminating health knowledge to others”, both the mean scores of the health behavior of the elderly in Henan were higher than those in Chongqing.

Different scores on the health knowledge and behaviors among the elderly in the sample areas were analyzed by using ANOVA. Table 4 shows a significant difference in the level of economic development (*p* = 0.000 < 0.001), that is, the increase in the level of economic development was in the following order: Chongqing < Henan < Zhejiang. A significant difference was also observed among the scores of the health behaviors of the elderly. The score in Zhejiang was higher than that in Chongqing and Henan. Given the significant difference in the results, the age groups of 60–65, 66–70 and 71–75 were observed to have higher health knowledge scores than the age groups of 76–80 and above 80 years old. A similar trend was observed in the scores of the health behaviors of the elderly.

The degree of education of the elderly resulted in significant differences in their health knowledge and behaviors (*p* = 0.000 < 0.001). The scores on the health knowledge and behaviors of illiterate elderly people and the elderly who graduated from primary school were lower than those of the elderly who graduated from junior high school, senior high school, and college. 

Table 4 shows that the different professions had different health knowledge and behavior scores (*p* = 0.000 < 0.001). The health knowledge scores of the migrant-workers were higher than those of the farmers and retirees. The health knowledge scores of the self-employed were higher than those of the retirees. The scores of the health behavior of migrant-workers were lower than those of the self-employed, retirees, and farmers. The results showed no significant differences in the health knowledge scores of the three types of healthcare insurance, whereas a significant difference was observed in the health behavior scores (*p* = 0.000 < 0.001). The score of UEBMI was higher than that of NCMS. A significant difference was determined in the item of distance from home to the medical institutions (*p* = 0.000 < 0.001). The longer the distance from home to medical institutions, the less health knowledge the elderly had. Both scores of health knowledge and behavior had significant differences with DPI (*p* = 0.000 < 0.001). The elderly with DPI of over 20,000 RMB, had higher health knowledge and behavior scores.

The factors that affect the health knowledge and behaviors of the elderly were analyzed by applying the multiple-linear regression method in Table 5. In terms of economic development, Zhejiang exhibited a significant difference with Chongqing, which was the reference sample area (*p* = 0.002 < 0.001). In terms of educational degree, with illiteracy as the reference, significant differences were observed between the group with illiteracy and groups with degrees from junior high school, senior high school, and college (*p* = 0.000 < 0.001). In terms of profession, with farmers as the reference, significant differences were observed between farmers and migrant-workers (*p* = 0.000 < 0.001), and between farmers and the self-employed (*p* = 0.000 < 0.001). A difference was observed in the item on distance from home to medical institutions (*p* = 0.000 < 0.001). In view of the DPI of the elderly in the sample areas, with those who earned less than 5000 RMB in DPI as a reference, those who earned more than 20,000 RMB in DPI has a difference with the reference (*p* = 0.001 < 0.01). 

## 4. Discussion

The aging condition throughout China is becoming increasingly serious because the increasing number of aging people is seen to be a key societal problem [37,38]. The majority of the sample elderly are illiterate. This observation represents the low level of economic development that caused the elderly to have limited opportunities to study in the past. Furthermore, they lacked learning awareness, which also causes illiteracy [39]. The survey results indicated that the majority of the elderly were farmers. Other professions, such as the self-employed, and migrant-workers are few. This finding indicated that few elderly people have professional skills, and lack basic knowledge to perform other types of work [40]. In China, particularly in the rural areas, farmers comprise more than 70% of the population, and the majority of these farmers choose NCMS, because this type of insurance can provide them with the largest proportion of reimbursement. Moreover, farmers are satisfied with the quality and practical benefits of NCMS. The Chinese scholar Wang analyzed the relations and level of satisfactions of farmers’ toward NCMS [41]. Regarding DPI, the respondents who had more than 20,000 RMB and less than 5000 RMB were the highest among the five types of DPI. The results indicated the existence of a serious gap between the rich and poor residents in the sample areas. The economic level of Zhejiang (Eastern China) is higher than that of Henan (Central China) and Chongqing (Western China). Under these circumstances, DPI should be increased to fit the level of economic development. Previous studies have suggested that lower health literacy is more prevalent among people with low levels of education and incomes and those who are older [42], demonstrating that DPI is an important factor affecting health behaviors of the elderly.

Individuals with limited health literacy have minimal health knowledge, poor self-management skills, low use of preventive services, and high hospitalization rates [43]. In general, health knowledge can influence an individual’s health behavior to a certain extent, and both play important roles in the health literacy of the elderly [44]. The highest accuracy rate of item 6, which asks “whether fruits can be eaten directly without washing” indicated that the elderly, even those without substantial health knowledge, possess basic health common sense. In addition, the elderly in the sample areas also aware of how they could protect themselves from infectious diseases and pesticide residue [45]. The accuracy rate of item 9, which asks “whether being healthy is defined as not being too fat or too thin” was the lowest. This finding indicated that the elderly in these areas had low levels of health knowledge, and lacked understanding of basic health knowledge. The results may be a cause for alarm, because some studies indicated that people with high health knowledge scores had good and healthy physical and mental states [46]. 

The accuracy rates of nearly all health knowledge items for Zhejiang were higher than those in Chongqing and Henna. Similar studies showed that the living environment, level of economic development and the comprehensive quality of the population can determine the health knowledge of individuals to a certain extent [47]. In contrast, the accuracy rates of the elderly in Chongqing and Henan were the lowest among the three sample areas. Low level of economic development and difficult environment factors lead to minimal health knowledge transmission among the population, particularly among the elderly, in the mountainous areas of Western and Central China, particularly in Western China, such as Chongqing.

Other studies have indicated that economic and environmental factors generally affect the health behaviors of the elderly [48,49]. In terms of the mean score of the elderly in the sample areas, significant differences were observed among most of the eleven items on health behaviors. This phenomenon showed that different areas in China have different economic development status, living environments, and customs, leading to different health behaviors among people. The results showed that no significant difference exists in the mean scores of the item asking about the “frequency of tooth brushing”. A possible reason for these results is the continuous improvement of economic conditions leading to an increased number of rural elderly people who can afford toothbrushes and toothpastes. The dental conditions of an individual are also a reflection of one’s physical health condition [50]. 

A significant difference was observed in item 3 asking about the “number of cigarettes smoked per day” (*p* = 0.002 < 0.001). The mean score of this item was observed to decrease in the following order: Zhejiang > Henan > Chongqing. The results indicated that majority of the respondents possibly possess healthy living consciousness and attitudes as well as that majority of the elderly who have quit smoking are aware of the harmful effects of smoking [51]. In terms of physical examinations, the elderly in Zhejiang (Eastern China) had the highest mean score. However, the mean scores of the elderly in Chongqing (Western China) were higher than that in Henan (Central China). This phenomenon demonstrates that an increased number of elderly people may be willing to go out for physical exercises. However, Chongqing is located in a mountainous areas and the climate causes many elderly people to suffer chronic diseases. Thus, the elderly in Chongqing need more physical examinations than those in Henan, which is located in the plains. The literature also showed that to a certain extent, the desire of an individual to engage in physical exercises is related to their living environment [52]. The MacArthur studies on “Successful Aging” also indicated that individuals who engage in volunteer activities have high physical and cognitive functioning [53]. 

Previous results showed that the mean score of the frequency of blood pressure measurement is the highest in Zhejiang, whereas Henan had lower mean score than in Chongqing. This finding indicated that many elderly people in Zhejiang (Eastern China) can purchase their own sphygmomanometer because they can afford it, thereby enabling them to test their blood pressure at any given time. Our survey was conducted in remote rural areas in Henna. Most elderly people cannot afford sphygmomanometers, and they also lacked health awareness, which resulted in lesser frequency of blood pressure measurement than in Chongqing. A previous studies has showed approximately 80% of American population aged 65 and above suffered from at least one chronic disease. In rural China, most elderly people did not know what the normal blood pressure rate was, and let alone how to prevent hypertension [54]. In addition, the mean score of health behavior in the item regarding the “frequency of disseminating health knowledge to others” in Henan was higher than in Chongqing. This finding indicated that people had easier access to health information in Eastern and Central China than in Western China. The reason may be attributed to the more convenient traffic conditions and flat terrain in Eastern and Central China [54,55,56]. 

The results in Table 4 show that a significant difference can be observed in levels of economic development. A good method that can be used is the “poverty” hypothesis, wherein ill-health is a consequence of lower income. A higher income has a significant contribution to health improvement among low income residents compared with high income residents. Hence, the level of economic development is associated with the state of people’s health [30]. A significant difference was observed among the age groups. In particular, the younger groups had higher scores than older ones among the sample elderly. This finding implied that age is related to the degree of health knowledge [53]. Further evidence showed that a positive effect could be observed between the degree of education and health quality, that is, the greater the health knowledge is, the better the health behaviors and quality of health [57,58]. 

Given that the score of UEBMI was higher than that of NCMS, certain elderly people who worked in urban areas but live in rural areas might have abundant knowledge on health [50]. Table 4 and Table 5 show that significant differences could be observed determined with regard to the distance from home to medical institutions. The results indicated that geographic conditions can determine the frequency of health guidance of village doctors to a certain extent. The fewer the chances an individual has to visit village doctors correspond to the reduction in their health knowledge, which affected their living behavior in return [59]. The elderly with DPIs higher than 20,000 RMB had higher health knowledge and health behavior scores. This phenomenon indicated that those with high income had more access to health knowledge and focus on to pay attention to their own health behaviors [58]. In 1993, Feinstein also determined the positive correlation between income and health behaviors [59]. 

## 5. Conclusions

Health knowledge and behaviors are both considered as important elements to improve the health status of the elderly. Furthermore, understanding the relative factors that affect health knowledge and behaviors is helpful in accessing, realizing, appraising, and applying health information. Few studies have studied the factors affecting health knowledge and health behaviors simultaneously. In this study, we explored the factors that affected the health knowledge, and health behaviors of the elderly and identified important factors such as age, degree of education, profession, types of healthcare insurance, distance from home to medical institutions, and DPI. 

In general, the scores of health knowledge and health behaviors of the elderly in Zhejiang are higher than those in Chongqing and Henan, which have relatively close economic development level. The health awareness of the elderly needs to be strengthened. Simultaneously, more focus should be given to the health knowledge and health behavior of the elderly. Furthermore, the government, society, medical institutions, and health workers should collaborate to help the elderly to promote their health condition and awareness in rural China. 

Although, this study has several limitations. We only examined the health knowledge and behaviors of the elderly in rural China because of our previous project design. Next, the sample sizes of the rural elderly population were taken from only three areas in China. Thus, these samples might not represent the conditions of all the elderly in China. Moreover, we did not compare the elderly in rural areas with the elderly in urban areas because of our original study design. In future studies, we could consider investigating the elderly in urban areas, which will compare with the elderly in rural China. 

## Figures and Tables

**Table 1:** Sampling strategy and valid sample size by area.

Municipality/Province	Towns	Villages & Total Sample Size	Sample Size (the Elders %)
Chongqing (western)	4 towns	16 villages & 576	533 (92.53)
Henan (central)	4 towns	16 villages & 576	522 (90.63)
Zhejiang (eastern)	4 towns	16 villages & 576	538 (93.40)

**Table 2:** Description of the socio-demographic characteristics of the elderly in sample areas.

Variables	Total		Chongqing		Henan		Zhejiang		χ^2^	*p*
	*n* = 1593	%	*n* = 533	%	*n* = 522	%	*n* = 538	%		
**Age groups**										
60–65	201	12.62	67	12.57	46	8.81	88	16.36	96.39	0.000 ***
66–70	514	32.27	138	25.89	161	30.84	215	39.96		
71–75	390	24.48	119	22.33	131	25.10	140	26.02		
76–80	321	20.15	120	22.51	118	22.61	83	15.43		
Above 80	167	10.48	89	16.70	66	12.64	12	2.23		
**Gender**										
Male	826	51.85	239	44.84	287	54.98	300	55.76	15.84	0.000 ***
Female	767	48.15	294	55.16	235	45.02	238	44.24		
**Degree of education**										
Illiteracy	811	50.91	285	53.47	258	49.43	268	49.81	24.10	0.002 ***
Primary school	516	32.39	187	35.08	158	30.27	171	31.78		
Junior high school	212	13.31	47	8.82	82	15.71	83	15.43		
Senior high school	37	2.32	10	1.88	13	2.48	14	2.61		
College	17	1.07	4	0.75	11	2.11	2	0.37		
**Profession**										
Farmer	1189	74.63	494	92.68	328	62.84	367	68.22	203.19	0.000 ***
Migrant-workers	74	4.65	3	0.56	60	11.49	11	2.05		
The self-employed	56	3.52	3	0.56	32	6.13	21	3.90		
Retirees	184	11.55	24	4.51	71	13.60	89	16.54		
Others	90	5.65	9	1.69	31	5.94	50	9.29		
**Types of healthcare insurance**										
NCMS	1475	92.59	502	94.18	495	94.83	478	88.85	19.40	0.001 ***
URBMI	53	3.33	17	3.19	13	2.49	23	4.28		
UEBMI	65	4.08	14	2.63	14	2.68	37	6.87		
**Distance from home to medical institutions**										
<1 km	1340	84.12	401	75.23	424	81.23	515	95.72	89.01	0.000 ***
≥1 km	253	15.88	132	24.77	98	18.77	23	4.28		
**Disposable Personal Income (DPI: RMB yuan)**										
<5000	367	23.04	168	31.52	109	20.88	90	16.73	186.81	0.000 ***
5001–10,000	230	14.44	111	20.83	64	12.26	55	10.22		
10,001–15,000	346	21.72	160	30.02	91	17.43	95	17.66		
15,001–20,000	121	7.60	26	4.88	43	8.24	52	9.67		
>20,000	529	33.20	68	12.75	215	41.19	246	45.72		

Note: The *p* value was calculated by chi-square test, *** *p* < 0.001.

**Table 3:** Accuracy rate of the health knowledge and mean scores of the health behavior of the elderly in sample areas.

**Health Knowledge**						
**Items**	**Total (*n* = 1593)**	**a. Chongqing (*n* = 533)**	**b. Henan (*n* = 522)**	**c. Zhejiang (*n* = 538)**	**χ^2^**	***p***
	**Accuracy Rate (%)**	**Accuracy Rate (%)**	**Accuracy Rate (%)**	**Accuracy Rate (%)**		
1. Whether the secondhand smoke is harmful to health	1160 (72.82)	365 (68.48)	355 (68.01)	440 (81.78)	33.02	0.000 ***
2. Whether too much salt cause high blood pressure	1105 (69.37)	304 (57.04)	330 (63.22)	471 (87.55)	131.10	0.000 ***
3. Fat people are vulnerable in diabetes	919 (57.69)	217 (40.71)	302 (57.85)	400 (74.35)	124.10	0.000 ***
4. Whether people suffer from hepatitis B after eating together with hepatitis B patients	757 (47.52)	222 (41.65)	239 (45.79)	296 (55.02)	20.12	0.000 ***
5. Whether excessive drinking is harmful to the liver	1175 (73.76)	392 (73.55)	352 (67.43)	431 (80.11)	22.02	0.000 ***
6. Whether fruits can be eaten without washing	1331 (83.55)	451 (84.62)	400 (76.63)	480 (89.22)	31.22	0.000 ***
7. Whether vaccinate is protected from infectious disease	692 (43.44)	280 (52.53)	291 (55.75)	121 (22.49)	146.20	0.000 ***
8. Whether the anemia related with the iron-deficiency	572 (35.91)	136 (25.52)	230 (44.06)	206 (38.29)	41.42	0.000 ***
9. Whether health is defined as not being fat or thin	523 (32.83)	86 (16.14)	194 (37.16)	243 (45.17)	109.00	0.000 ***
Average accuracy	(57.43)	(51.14)	(57.32)	(63.78)		
**Health Behavior**						
**Items**	**Total (*n* = 1593)**	**a. Chongqing (*n* = 533)**	**b. Henan (*n* = 522)**	**c. Zhejiang (*n* = 538)**	***p***	**LSD**
	**Mean**	**Mean**	**Mean**	**Mean**		
1. Heavy taste	2.17	2.14	2.01	2.36	0.000 ***	c > a > b **
2. Frequency of tooth brushing	1.78	1.80	1.74	1.79	0.462	
3. Cigarette per day	2.45	2.36	2.43	2.57	0.002 **	c > a **; c > b **
4. Drinking per day	2.66	2.59	2.57	2.83	0.000 ***	c > a ***; c > b ***
5. Physical examination	1.77	1.58	1.37	2.35	0.000 ***	c > a > b **
6. Frequency of physical exercise	1.19	0.87	1.02	1.68	0.000 ***	c > b > a **
7.Purchase times of expired food	2.55	2.48	2.40	2.75	0.000 ***	c > a ***; c > b ***
8. Frequency of blood pressure measurement	1.69	1.52	1.39	2.14	0.000 ***	c > a > b **
9. Frequency of obtaining health knowledge	1.61	1.33	1.42	2.06	0.000 ***	c > a ***; c > b ***
10. Frequency of consulting health knowledge from doctors	1.85	1.63	1.69	2.23	0.000 ***	c > a ***; c > b ***
11. Frequency of disseminating health knowledge to others	0.70	0.43	0.59	1.07	0.000 ***	c > b > a **

Notes: ** *p* < 0.01, *** *p* < 0.001.

**Table 4:** Scores of health knowledge and health behavior under different items of the elderly in sample areas.

Characteristic Variables	n	Scores of Health Knowledge					Scores of Health Behavior				
		Mean	SD	F	*p*	LSD	Mean	SD	F	*p*	LSD
a. Chongqing	533	4.60	2.29	31.52	0.000 ***	a < b < c ***	18.74	4.28	360.01	0.000 ***	c > a ***; c > b ***
b. Henan	522	5.16	2.75				18.63	3.64			
c. Zhejiang	538	5.74	1.94				23.86	2.88			
a. 60–65	201	5.54	2.07	19.29	0.000 ***	a > d > e **; b > d > e **; c > d > e **	20.85	4.11	24.65	0.000 ***	a > d > e **; b > d > e **; c > d > e **
b. 66–70	514	5.40	2.33				21.23	4.35			
c. 71–75	390	5.43	2.36				20.88	4.39			
d. 76–80	321	4.98	2.42				19.73	4.28			
e. over 80	167	3.77	2.46				17.76	3.81			
a. Male	826	5.17	2.38	0.00	0.991		20.33	4.39	0.91	0.34	
b. Female	767	5.17	2.40				20.54	4.38			
a. Illiteracy	811	4.96	2.49	15.56	0.000 ***	a < c ***; a < c ***; a < d **; b < c ***; b < c ***; b < e **	20.03	4.40	8.61	0.000 ***	a < c ***; a < d ***; a < e **; b < c **; b < d **; b < e **
b. Primary school	516	4.99	2.23				20.39	4.30			
c. Junior high school	212	6.04	2.20				21.46	4.36			
d. Senior high school	37	6.59	1.64				22.76	4.03			
e. College	17	6.94	1.20				22.76	3.56			
a. Farmer	1189	4.97	2.31	15.00	0.000 ***	b > a ***; c > a ***; b > d ***; b > e ***; c > d **; c > e **	20.26	4.29	18.68	0.000 ***	b < a < d < e **; b < c ***
b. Migrant-worker	74	6.84	2.26				17.28	3.82			
c. The self-employed	56	6.30	2.32				21.34	4.10			
d. Retirees	184	5.29	2.70				22.01	4.36			
e. Others	90	5.41	2.22				21.50	4.72			
a. NCMS	1475	5.17	2.40	0.01	0.993		20.34	4.40	5.51	0.004 **	c > a **
b. URBMI	53	5.21	1.78				20.96	3.99			
c. UEBMI	65	5.17	2.61				22.11	4.18			
a. <1 km	1340	5.30	2.38	24.63	0.000 ***	a > b ***	20.86	4.30	87.40	0.000 ***	a > b ***
b. ≥1 km	253	4.49	2.34				18.13	4.14			
a. <5000 RMB	367	4.84	2.51	11.03	0.000 ***	e > a ***; e > b ***; e > c ***; e > d **	19.94	4.32	11.30	0.000 ***	d > a **; d > b **; d > c **; e > a ***; e > b ***; e > c ***
b. 5001–10,000 RMB	230	4.86	2.26				19.52	4.09			
c. 10,001–15,000 RMB	346	4.90	2.42				19.94	4.44			
d. 15,001–20,000 RMB	121	5.16	2.31				21.08	4.37			
e. >20,000 RMB	529	5.71	2.27				21.34	4.36			

Note: The *p* value was calculated by ANOVA analysis, ** *p* < 0.01, *** *p* < 0.001.

**Table 5:** Multiple−linear regression analysis of factors of health knowledge and behavior among the elderly in sample areas.

	Influencing Factors of Health Knowledge							Influencing Factors of Health Behaviors						
Characteristic Variables	Unstandardized Coefficients		Standardized Coefficients	*t* Value	*p*	95% Confidence		Unstandardized Coefficients		Standardized Coefficients	*t* Value	*p*	95% Confidence	
	B	SE	Beta			Lower	Upper	B	SE	Beta			Lower	Upper
Constant	5.515	0.285		19.343	0.000 ***	4.956	6.074	20.199	0.448		45.079	0.000 ***	19.320	21.078
Henan	−0.036	0.150	−0.007	−0.243	0.808	−0.330	0.257	−0.408	0.235	−0.044	−1.733	0.083	−0.869	0.054
Zhejiang	0.484	0.155	0.096	3.113	0.002 **	0.179	0.788	4.254	0.244	0.459	17.427	0.000 ***	3.775	4.733
66–70	−0.067	0.187	−0.013	−0.360	0.719	−0.433	0.299	0.406	0.293	0.043	1.383	0.167	−0.170	0.981
71–75	0.029	0.196	0.005	0.148	0.882	−0.355	0.413	0.436	0.308	0.043	1.417	0.157	−0.167	1.039
76–80	−0.311	0.203	−0.052	−1.528	0.127	−0.710	0.088	−0.218	0.320	−0.020	−0.682	0.495	−0.845	0.409
Above 80	−1.278	0.242	−0.164	−5.274	0.000 **	−1.753	−0.803	−1.324	0.381	−0.092	−3.477	0.001 **	−2.071	−0.577
Primary school	−0.005	0.127	−0.001	−0.043	0.966	−0.254	0.243	0.184	0.199	0.020	0.924	0.356	−0.207	0.575
Junior high school	0.914	0.175	0.130	5.214	0.000 ***	0.570	1.258	1.151	0.275	0.089	4.179	0.000 ***	0.611	1.691
Senior high school	1.529	0.376	0.096	4.066	0.000 ***	0.792	2.267	2.182	0.591	0.075	3.693	0.000 ***	1.023	3.341
College	1.882	0.551	0.081	3.417	0.001 **	0.802	2.963	3.267	0.866	0.077	3.773	0.000 ***	1.569	4.965
Migrant-workers	1.689	0.279	0.149	6.062	0.000 ***	1.143	2.236	−2.259	0.438	−0.108	−5.157	0.000 ***	−3.118	−1.400
The self-employed	1.144	0.310	0.088	3.686	0.000 ***	0.535	1.753	0.810	0.488	0.034	1.660	0.097	−0.147	1.767
Retirees	−0.099	0.183	−0.013	−0.544	0.587	−0.457	0.259	0.651	0.287	0.047	2.270	0.023 *	0.088	1.214
Others	0.282	0.249	0.027	1.135	0.257	−0.206	0.771	0.158	0.391	0.008	0.404	0.686	−0.609	0.925
Distance from home to healthcare institutions	−0.675	0.158	−0.103	−4.263	0.000 ***	−0.986	−0.364	−1.280	0.249	−0.107	−5.146	0.000 ***	−1.768	−0.792
5001–10,000 RMB	0.047	0.188	0.007	0.249	0.803	−0.322	0.416	−0.332	0.296	−0.027	−1.122	0.262	−0.911	0.248
10,001–15,000 RMB	−0.001	0.168	0.000	−0.008	0.994	−0.331	0.329	−0.135	0.264	−0.013	−0.510	0.610	−0.653	0.384
15,001–20,000 RMB	0.167	0.236	0.019	0.708	0.479	−0.296	0.630	0.348	0.371	0.021	0.938	0.348	−0.380	1.076
>20,000 RMB	0.536	0.158	0.106	3.382	0.001 **	0.225	0.847	0.306	0.249	0.033	1.230	0.219	−0.182	0.795

Note: The *p* value was calculated by ANOVA analysis, * *p* < 0.05, ** *p* < 0.01, *** *p* < 0.001.

## Abbreviations

The following abbreviations are used in this manuscript:

DPI	Disposable personal income
NCMS	New rural cooperative medical system
UEBMI	Urban Employee Basic Medical Insurance
URBMI	Urban Residents Basic Medical Insurance
